# Association between immune check point inhibitors and digestive system inflammatory adverse reactions: evidence from pharmacovigilance analysis and systematic review

**DOI:** 10.3389/fphar.2025.1684475

**Published:** 2025-10-27

**Authors:** Ya Zou, Qinchuan Li, Lu Zhou, Yun Lu, Hua Wei, Yan Zhou, Shibo Lin, Xirui Guo, Shihao Yan, Hongju Wang, Fangqing Xie, Chun Liu, Li Chen

**Affiliations:** ^1^ Department of Clinical Pharmacology, Chengdu Second People’s Hospital, Chengdu, China; ^2^ Department of Pharmacy and Evidence Based Pharmacy Center, West China Second University Hospital, Sichuan University, Chengdu, China Department of Pharmacology, Faculty of Medicine, University of the Basque Country, UPV/EHU, Leioa, Spain

**Keywords:** ICI, digestive inflammatory adverse reactions, data mining, pharmacovigilance, sialadenitis

## Abstract

**Purpose:**

Comparative real-world data on the spectrum of digestive inflammatory adverse reactions across ICI classes are limited. Existing evidence on immune-related Sjögren’s syndrome/sialadenitis consists largely of case reports and small series.

**Methods:**

We performed disproportionality analysis using the FDA Adverse Event Reporting System (FAERS) database (2015–2023) to evaluate associations between ICIs and digestive inflammatory adverse reactions. Additionally, we conducted a systematic review up to July 2025 to identify published cases of ICI-associated Sjögren’s syndrome/sialadenitis.

**Results:**

PD-1 inhibitors (pembrolizumab and nivolumab) showed the strongest associations with immune-mediated oesophagitis and gastritis. Pembrolizumab was also highly associated with hepatobiliary events, including immune-mediated cholangitis (ROR 249.18, 95% CI 169.04-367.32) and hepatitis (ROR 85.51, 95% CI 73.22-99.86). In contrast, the CTLA-4 inhibitor ipilimumab exhibited the strongest signal for immune-mediated enterocolitis. Atezolizumab and ipilimumab were significantly associated with spontaneous bacterial peritonitis. Our systematic review identified 93 cases of ICI-associated Sjögren’s syndrome/sialadenitis, predominantly in patients with melanoma or lung cancer receiving PD-1 inhibitors.

**Conclusion:**

PD-1 inhibitors are more strongly associated with upper GI and hepatobiliary inflammatory adverse reactions, whereas CTLA-4 inhibitors carry a higher risk of enterocolitis. These findings underscore the need for ICI-specific monitoring protocols. Early recognition and tailored management—including potential treatment interruption or corticosteroid use—are critical to minimizing severe outcomes. Clinicians should maintain a high index of suspicion for rare inflammatory adverse reactions such as sialadenitis, even as incidence remains low. These insights support more personalized risk-benefit assessment and inflammatory adverse reactions management in patients receiving ICIs.

## 1 Introduction

Over the past decade, immune checkpoint inhibitors (ICIs) have become pivotal antineoplastic agents, now serving as first- or second-line therapies for malignancies such as non-small cell lung cancer, head and neck squamous cell carcinoma, gastric or gastroesophageal junction adenocarcinoma, colorectal cancer, and melanoma (Qingli et al., 2023; Boutros et al., 2023; Fan et al., 2023). By targeting immune regulatory molecules including PD-1, PD-L1, and CTLA-4, ICIs enhance T cell-mediated antitumor immunity. However, this activity can also induce inflammatory immune-related adverse events (irAEs), with the gastrointestinal tract being one of the most commonly affected systems (Tan et al., 2020; Nicholls et al., 2023; Wei and Luo, 2017).

While colitis and hepatitis are well-documented (Gong and Wang, 2020; Wang et al., 2018; Wang DY. et al., 2017), other digestive system irAEs—such as pancreatitis, cholangitis, gastritis, sialadenitis, oesophagitis, cholecystitis, and peritonitis—remain understudied. Existing clinical trials are often limited by small sample sizes and short follow-up periods, hindering a comprehensive understanding of the full spectrum and impact of these events (Pulini et al., 2021; Alomar et al., 2019). Although prior analyses of the FDA Adverse Event Reporting System (FAERS) database have offered valuable insights into certain irAEs (Moore et al., 2024; Milutinovic et al., 2024; Larkin et al., 2015; Khoja et al., 2017), a systematic evaluation covering both common and rare digestive system inflammatory adverse reactions across multiple ICIs is still lacking.

To address this gap, this study conducts a comprehensive pharmacovigilance analysis using the FAERS database to characterize the reporting patterns, clinical features, and risk profiles of a broad range of ICI-associated digestive system inflammatory adverse reactions. Focusing particularly on underreported conditions such as sialadenitis, oesophagitis, cholecystitis, and peritonitis—in comparison to colitis and hepatitis—our findings aim to provide clinicians and researchers with a clearer epidemiological and risk-assessment framework to support the safe and rational use of ICIs in oncology.

## 2 Materials and methods

### 2.1 Pharmacovigilance analysis

#### 2.1.1 Data sources

This retrospective pharmacovigilance study was conducted based on the FAERS database. The FAERS database includes the following eight types of files: demographic and administrative information (DEMO), drug information (DRUG), adverse events (REAC), patient outcomes (OUTC), report sources (RPSR), start and end dates for reported drugs (THER), indications for use (INDI), and invalid reports (DELETED). All files recorded “primaryid” and “caseid” variables; therefore, the information about patients and AEs could be obtained by linking these variables in all files.

All reports between 1 January 2015 and 31 December 2023 were extracted for this analysis. We chose 2015 as the starting year because Ipilimumab was first marketed in 2011, while the other four were launched after 2014. The study period was also the data analysis period, given that our research was a cross-sectional study.

#### 2.1.2 Data extraction

The generic and brand names of ICI approved by the FDA, including pembrolizumab (KEYTRUDA), atezolizumab (TECENTRIQ), durvalumab (IMFINZI), nivolumab (OPDIVO), and ipilimumab (YERVOY), were used to identify adverse events associated with ICI in the DRUG files.

The AEs in REAC files are encoded by the Preferred Terms (PTs) in the Medical Dictionary for Regulatory Activities (MedDRA) terminology, which comprises 27 system organ classes (SOCs) (Omar et al., 2021). The structural hierarchy of the MedDRA terminology has five levels: SOC (system organ class), HLGT (high-level group term), HLT (high-level term), PT (preferred term), and LLT (lowest level term). Accordingly, the latest version of MedDRA 27.0 was used to classify AEs in reports at the relevant SOC level. Our study included only cases where the target ICIs (pembrolizumab, atezolizumab, durvalumab, nivolumab and ipilimumab) were listed as primary suspects, while “secondary suspicion”, “concurrent medication”, and “interaction” were excluded. Inflammatory adverse reactions of all digestive system manifestations studied are shown in Supplementary Table S1 (Supplementary Material 1) (Zhou, 2003; Zhang and Zheng, 2015).

Demographics (gender, age), reporting characteristics (reporting country, year, occupation of reporters), and signal values of reports of ICIs-associated digestive system irAEs were analyzed.

#### 2.1.3 Data mining

All characteristics of the irAE reports regarding ICIs were evaluated descriptively. Categorical variables are reported as frequencies and percentages, and continuous variables are summarized as means with standard deviations (SD) or medians with interquartile ranges (IQR) based on data distribution.

Based on the fourfold table of proportion imbalance method (Table 1), the target ADE report number and ADE occurrence background number of the primary suspected drug were obtained.

**TABLE 1:** Fourfold table for disproportionality analyses.

Event groups	Drug used	Other drugs	Sums
Event	a	c	a + c
Other events	b	d	b + d
Sums	a + b	c + d	a + b + c + d

Then, a disproportionality analysis model was used to detect the potential signals of irAEs caused by ICIs at both the class level and the generic drug name level (Naida et al., 2021).

When a specific drug demonstrates a stronger association with a particular irAE compared to other medications, it typically yields a higher disproportionality score, reflecting its increased reporting frequency. Both frequentist methods (reporting odds ratio [ROR] and proportional reporting ratio [PRR] of disproportionality analysis) were applied to investigate the association between irAEs and ICIs (Robert et al., 2015). The equations and corresponding criteria of the two disproportionality algorithms are listed in Table 2.

**TABLE 2:** Summary of major algorithms used for signal detection.

Algorithms	Equation	Criteria
ROR	ROR = ad/bc	95%CI > 1, N ≥ 3
	95%CI = e^ln(ROR)±1.96(1/a+1/b+1/c+1/d)^0.5^	
PRR	PRR = a (c + d)/c/(a + b)	PRR ≥ 2, χ^2^ ≥ 4, N ≥ 3
	χ^2^ = [(ad − bc)^2^](a + b + c + d)/[(a + b) (c + d) (a + c) (b + d)]	

95%CI > 1 is the lower limit of the 95% CI > 1.

As shown in Supplementary Table S1 (Supplementary Material 1), we used the keywords sialoadenitis, oesophagitis, hepatitis, cholecystitis, cholangitis, peritonitis, pancreatitis, gastritis, and intestinal inflammation to identify inflammatory adverse reactions associated with the digestive system. Furthermore, for a signal to be considered significant, it had to meet all the following criteria concurrently for each algorithm. For ROR: The lower limit of the 95% confidence interval (95% CI) must be greater than 1, and the number of cases (N) must be at least 3. For PRR: The PRR point estimate must be greater than or equal to 2, the chi-squared (χ^2^) value must be at least 4, and the number of cases (N) must be at least 3. These predefined criteria, as detailed in Table 2, were applied consistently across all analyses at both the drug class level (all ICIs combined) and the individual generic drug name level.

#### 2.1.4 Statistical analysis

The demographic and clinical characteristics of patients experiencing ICI-associated digestive system irAEs were analyzed using descriptive statistics. Categorical variables are presented as numbers (n) and percentages (%). Normally distributed data are expressed as mean ± standard deviation (SD), while non-normally distributed data are expressed as median with interquartile range (IQR).

The proportions of patients with irAEs associated with different ICIs were compared using Fisher’s exact test or Pearson’s chi-squared test. Two-sided P values <0.05 were considered to indicate statistical significance.Data analysis was performed via SPSS 29.0 (IBM, Armonk, NY, USA)) and GraphPad Prism 10 (GraphPad Software, CA, USA).

### 2.2 Systematic review

#### 2.2.1 Search strategy

The retrospective case series was conducted in accordance with the STROBE (Strengthening the Reporting of Observational Studies in Epidemiology) guidelines and utilized the literature search strategy detailed in Supplementary Table S2 (Supplementary Material 1). It is worth noting that, this study utilized PubMed’s MeSH vocabulary for subject term searches. This feature automatically expands the search scope to include all sub-terms and related concepts under the target MeSH terms, ensuring comprehensive coverage and reducing omission bias. For example, searching’ Sjögren’s Syndrome’ [Mesh] automatically includes literature terms like ‘xerophthalmia’ and ‘xerostomia’. We systematically retrieved articles from PubMed covering the period from database inception to July 2025. Following manual verification of included studies, we identified additional eligible research and conducted analyses exclusively on English-language published literature. However, the limitation of our systematic review is that the database searched is limited to PubMed because relevant case reports may be indexed by EMBASE, Scopus or Web of Science.

#### 2.2.2 Selection criteria

Our study included the following types of studies: case reports and case series. Exclusion criteria included: observational studies, randomized controlled trials (RCTs), review articles, letters and correspondence involving relevant cases, meta-analyses, duplicate cases, review articles lacking patient information, conference abstracts, and animal experiments. Inclusion criteria were: 1) Studies containing individual case reports or case series; 2) Patients diagnosed with immune checkpoint inhibitor-related Sjögren-like syndrome/sialadenitis. In addition, there was a limitation that a critical evaluation tools (e.g., CARE checklist) were not used to evaluate the quality of included case reports.

The National Comprehensive Cancer Network (NCCN) currently does not have independent diagnostic criteria for ICI-related Sjogren-like syndrome or sialadenitis. For each included study, we extracted data regarding the diagnostic approach used to confirm the adverse event. We evaluated and recorded whether the study explicitly stated the use of, or reported findings that met, the formal diagnostic criteria outlined in Table 3. This was not used as an exclusion criterion but rather to assess the diagnostic rigor and comparability across the included case reports.

**TABLE 3:** Diagnostic criteria for immune checkpoint inhibitors-induced Sjögren’s syndrome/sialadenitis.

① New-onset symptoms of dry mouth (xerostomia), dry eyes (keratoconjunctivitis sicca), or salivary gland enlargement following immune checkpoint inhibitor (ICI) therapy
② Abnormal salivary gland function tests, such as reduced salivary flow rate or abnormal findings on salivary gland scintigraphy
③ Possible positivity for anti-SSA/SSB antibodies on serological testing (primary Sjögren’s syndrome must be excluded)
④ Histopathological evidence of lymphocytic sialadenitis on biopsy (if biopsy is performed)
⑤ Other etiologies of Sjögren’s syndrome/sialadenitis were excluded

## 3 Results

### 3.1 FAERS database

#### 3.1.1 Descriptive analysis

From January 2015 to December 2023, a total of 358,419 ICIs-associated AE reports were recorded, of which 1,538 reports of digestive system inflammatory adverse reactions were identified. Of these, 541 reports of digestive system inflammatory adverse reactions were associated with pembrolizumab (35.18%), 286 with atezolizumab (18.60%), 69 with durvalumab (4.49%), 436 with nivolumab (28.35%), and 206 with ipilimumab (13.39%). The characteristics of irAEs reported for different ICIs are presented in Table 4. More male patients reported irAEs from ICI (54.32%). The region with the highest number of reports was North America (34.36%), followed by Asia (31.43%) and Europe (30.27%). The number of digestive system irAEs steadily increased from 1,258 in 2018 to 1,538 in 2023, which reflects the increasing clinical application of ICI. Most irAEs were mainly reported by health-professionals (69.82%).

**TABLE 4:** The characteristics of ICIs associated digestive system irAEs.

Characteristics	All ICIs (N = 1,538)	Pembrolizumab (N = 541)	Atezolizumab (N = 286)	Durvalumab (N = 69)	Nivolumab (N = 436)	Ipilimumab (N = 206)
Demographics						
Sex (%)						
Female	534 (34.7%)	190 (35.1%)	99 (34.6%)	24 (34.8%)	151 (34.6%)	70 (34.0%)
Male	876 (57.0%)	308 (56.9%)	163 (57.0%)	39 (56.5%)	248 (56.9%)	118 (57.3%)
Unknown	128 (8.3%)	43 (7.9%)	24 (8.4%)	6 (8.7%)	37 (8.5%)	18 (8.7%)
Age Group (%)						
<18	54 (3.5%)	19 (3.5%)	10 (3.5%)	-	15 (3.4%)	8 (3.9%)
18–44	231 (15.0%)	81 (15.0%)	43 (15.0%)	12 (17.4%)	65 (14.9%)	32 (15.5%)
45–64	738 (48.0%)	260 (48.1%)	137 (47.9%)	33 (47.8%)	209 (47.9%)	99 (48.1%)
65–74	369 (24.0%)	130 (24.0%)	69 (24.1%)	17 (24.6%)	105 (24.1%)	48 (23.3%)
≥75	123 (8.0%)	43 (7.9%)	23 (8.0%)	5 (7.2%)	35 (8.0%)	17 (8.3%)
Unknown	23 (1.5%)	8 (1.5%)	4 (1.4%)	2 (2.9%)	7 (1.6%)	2 (1.0%)
Reporter (%)						
Physician	1,077 (70.0%)	379 (70.1%)	200 (69.9%)	48 (69.6%)	305 (70.0%)	145 (70.4%)
Pharmacist	231 (15.0%)	81 (15.0%)	43 (15.0%)	10 (14.5%)	65 (14.9%)	32 (15.5%)
Lawyer	2 (0.1%)	1 (0.2%)	0 (0.0%)	0 (0.0%)	1 (0.2%)	0 (0.0%)
Consumer	77 (5.0%)	27 (5.0%)	14 (4.9%)	3 (4.3%)	22 (5.0%)	11 (5.3%)
Other Health Professional	123 (8.0%)	49 (9.1%)	29 (10.1%)	6 (8.7%)	35 (8.0%)	16 (7.8%)
Unknown	28 (1.8%)	4 (0.7%)	-	2 (2.9%)	8 (1.8%)	2 (1.0%)
Reporter Year (%)						
2015	57 (3.7%)	27 (5.0%)	-	-	22 (5.0%)	8 (3.8%)
2016	118 (7.7%)	44 (8.1%)	23 (8.0%)	-	35 (8.0%)	16 (7.8%)
2017	185 (12.0%)	65 (12.0%)	34 (11.9%)	8 (11.6%)	52 (11.9%)	26 (12.6%)
2018	231 (15.0%)	81 (15.0%)	43 (15.0%)	10 (14.5%)	65 (14.9%)	32 (15.5%)
2019	277 (18.0%)	97 (17.9%)	52 (18.2%)	13 (18.8%)	78 (17.9%)	37 (18.0%)
2020	331 (21.5%)	108 (20.0%)	71 (24.8%)	23 (33.3%)	87 (20.0%)	42 (20.4%)
2021	231 (15.0%)	81 (15.0%)	43 (15.0%)	10 (14.5%)	65 (14.9%)	32 (15.5%)
2022	62 (4.0%)	22 (4.1%)	12 (4.2%)	3 (4.3%)	18 (4.1%)	7 (3.4%)
2023	46 (3.0%)	16 (3.0%)	8 (2.8%)	2 (2.9%)	14 (3.2%)	6 (2.9%)
Region (%)						
Asian	461 (30.0%)	162 (29.9%)	86 (30.1%)	21 (30.4%)	131 (30.0%)	61 (29.6%)
European	385 (25.0%)	135 (25.0%)	72 (25.2%)	17 (24.6%)	109 (25.0%)	52 (25.2%)
South American	123 (8.0%)	43 (7.9%)	23 (8.0%)	6 (8.7%)	35 (8.0%)	16 (7.8%)
North American	506 (32.9%)	180 (33.3%)	95 (33.2%)	22 (31.9%)	139 (31.9%)	69 (33.5%)
Oceania	46 (3.0%)	16 (3.0%)	9 (3.1%)	2 (2.9%)	13 (3.0%)	6 (2.9%)
African	15 (1.0%)	5 (0.9%)	-	1 (1.4%)	4 (0.9%)	2 (1.0%)
Unknown	2 (0.1%)	-	1 (0.3%)	0 (0.0%)	5 (1.1%)	-

#### 3.1.2 Inflammatory adverse reactions signals associated with different ICI

**FIGURE 1:**
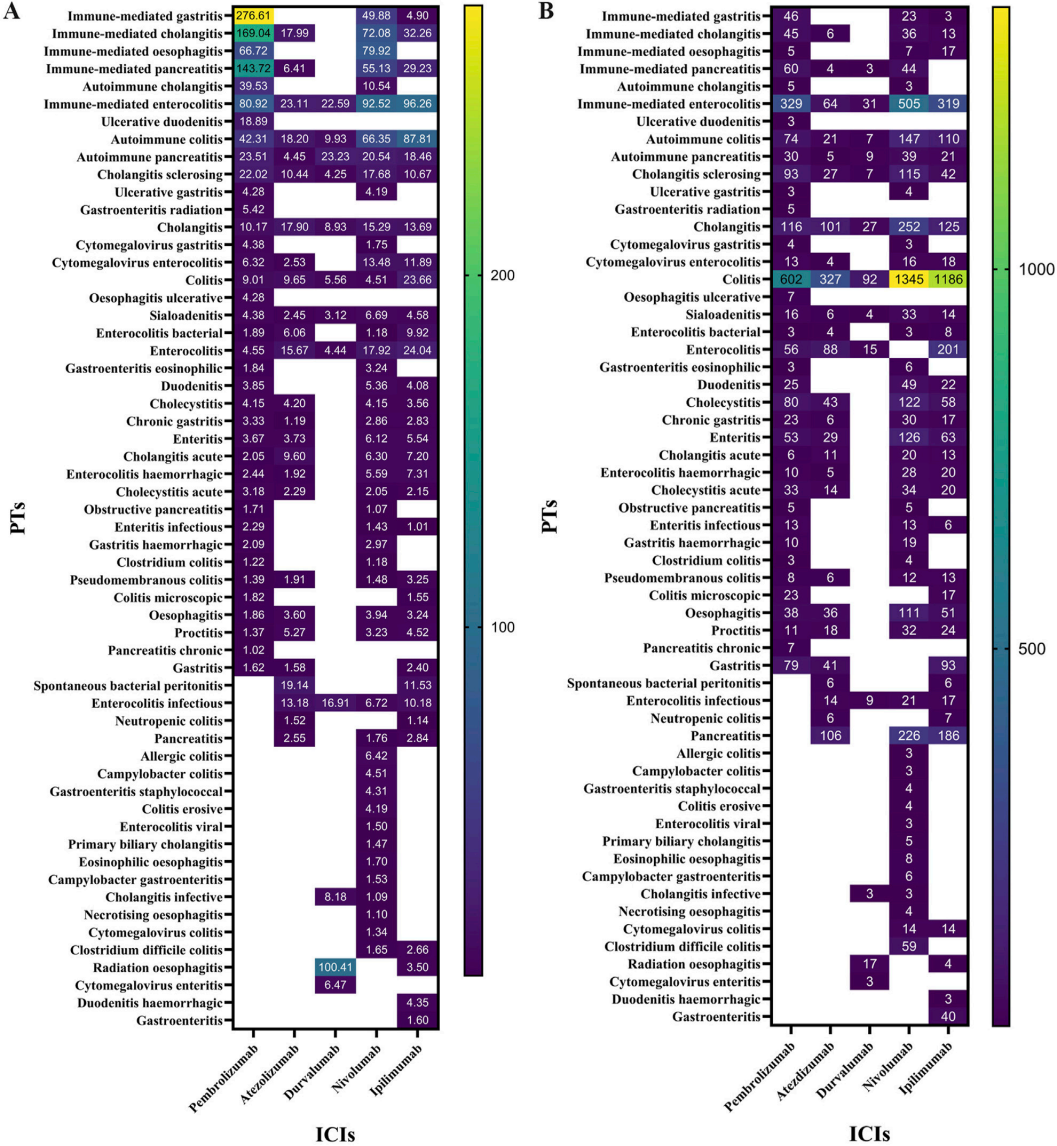
**(A)** ROR 95%Cl lower of all PTs associated with lCls. **(B)** N of all PTs associated with ICls.

All irAEs signals of ICI were detected by using two algorithms and their corresponding criteria. The positive signals of digestive system irAEs were classified as PT. After signal detection of all digestive system irAEs related to ICIs, we found digestive system inflammatory adverse reactions (e.g., colitis, gastritis, etc.) among the irAEs with the highest and most frequent disproportionality signals. A total of 159 positive signal for ICIs were observed, the top ten adverse events in terms of signal strength were all inflammatory adverse reactions. We visualized the lower limit of the 95% confidence interval of the ROR and number of all PTs associated with ICI using heatmaps (Figure 1).

Our disproportionality analysis identified strong and statistically significant safety signals for autoimmune colitis (ROR=108.41, 95% CI 87.81-133.84) with ipilimumab and immune-mediated hepatitis (ROR=85.51, 95% CI 73.22-99.86) associated with pembrolizumab in the FAERS database. These robust signals warrant further clinical investigation to characterize their real-world incidence and impact.

**FIGURE 2:**
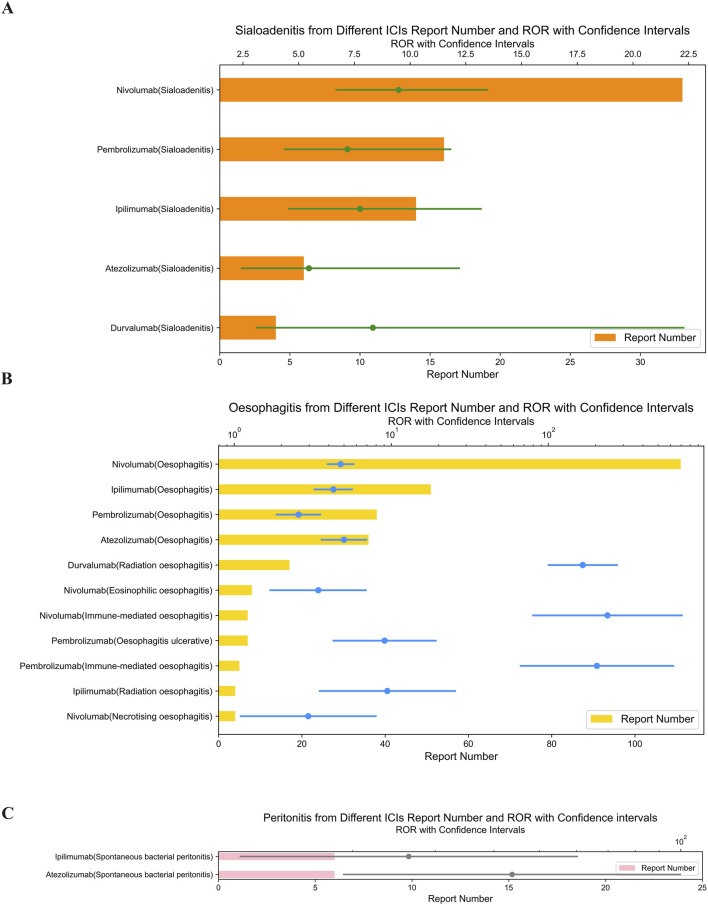
**(A)** Signals with sialoadenitis from different ICI. **(B)** Signals with oesophagitis from different ICI. **(C)** Signals with peritonitis from different ICI.

In Figure 2A, Nivolumab showed the highest signal for sialadenitis (ROR = 9.49; 95% CI: 6.69–13.46). Immune-mediated oesophagitis was reported with pembrolizumab (ROR = 203.97; 95% CI: 66.72–623.52) and nivolumab (ROR = 237.83; 95% CI: 79.92–707.71) (Figure 2B). It is important to note that the above results with wide confidence intervals should be considered as hypothetical generation rather than deterministic conclusions.

**FIGURE 3:**
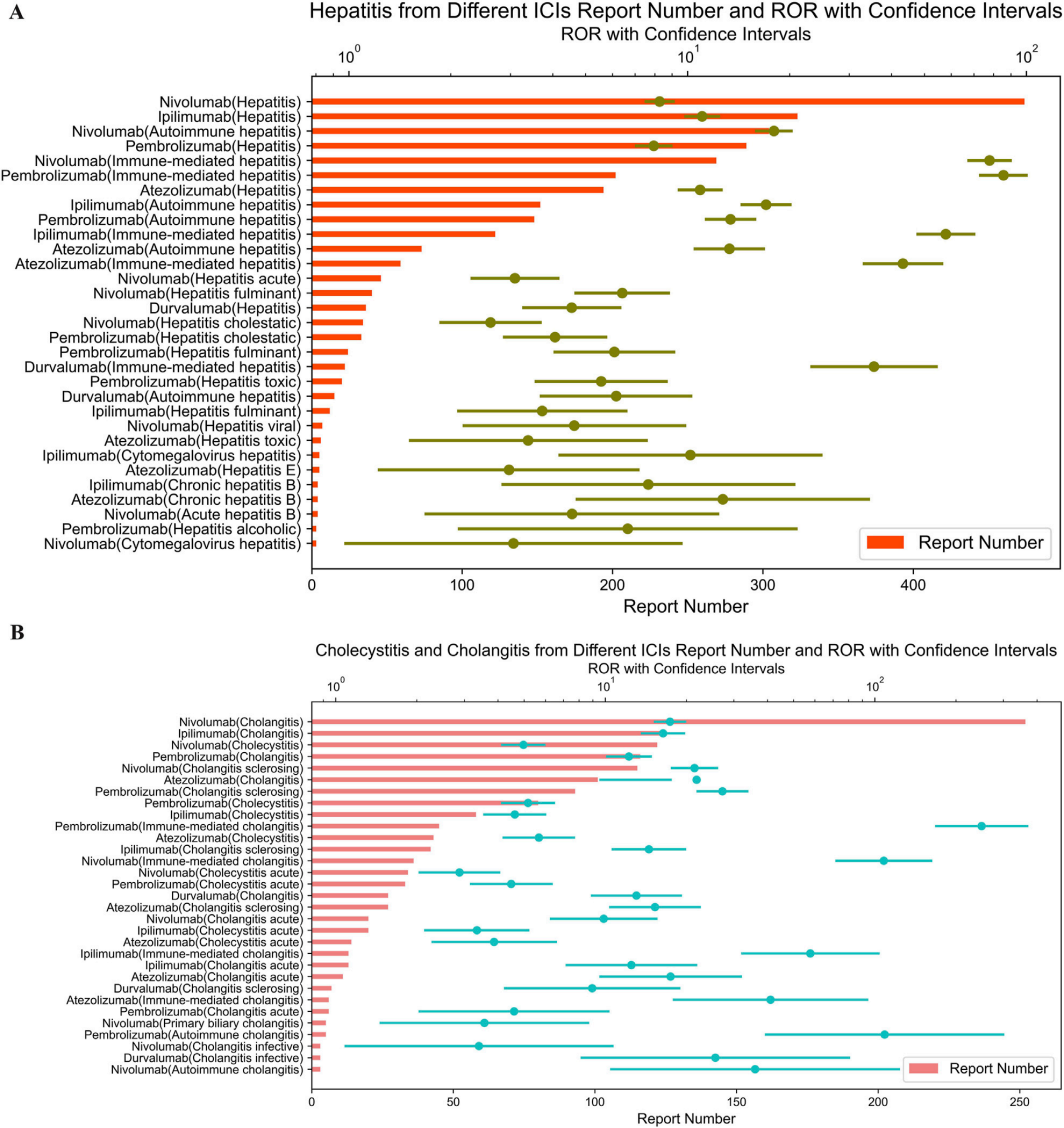
**(A)** Signals with gastritis from different ICI. **(B)** Signals with cholecystitis and cholangitis from different ICI.

Atezolizumab, an anti-PD-L1 drug, showed the highest signal strength for spontaneous bacterial peritonitis (ROR = 43.71; 95% CI: 19.14–99.83) (Figure 2C) and cholecystitis (ROR = 5.67; 95% CI: 4.20–7.65) (Figure 3B).

Similarly, as shown in Figure 3A, immune-mediated gastritis was associated with pembrolizumab (ROR = 429.39; 95% CI: 276.61–666.55), nivolumab (ROR = 80.86; 95% CI: 49.88–131.07), and ipilimumab (ROR = 15.52; 95% CI: 4.90–49.19). Meanwhile, we have compiled all 2 × 2 contingency tables (including a, b, c, d values) for ICIs and oesophagitis/gastritis event combinations into new supplementary tables (Supplementary Tables S4, S5).

**FIGURE 4:**
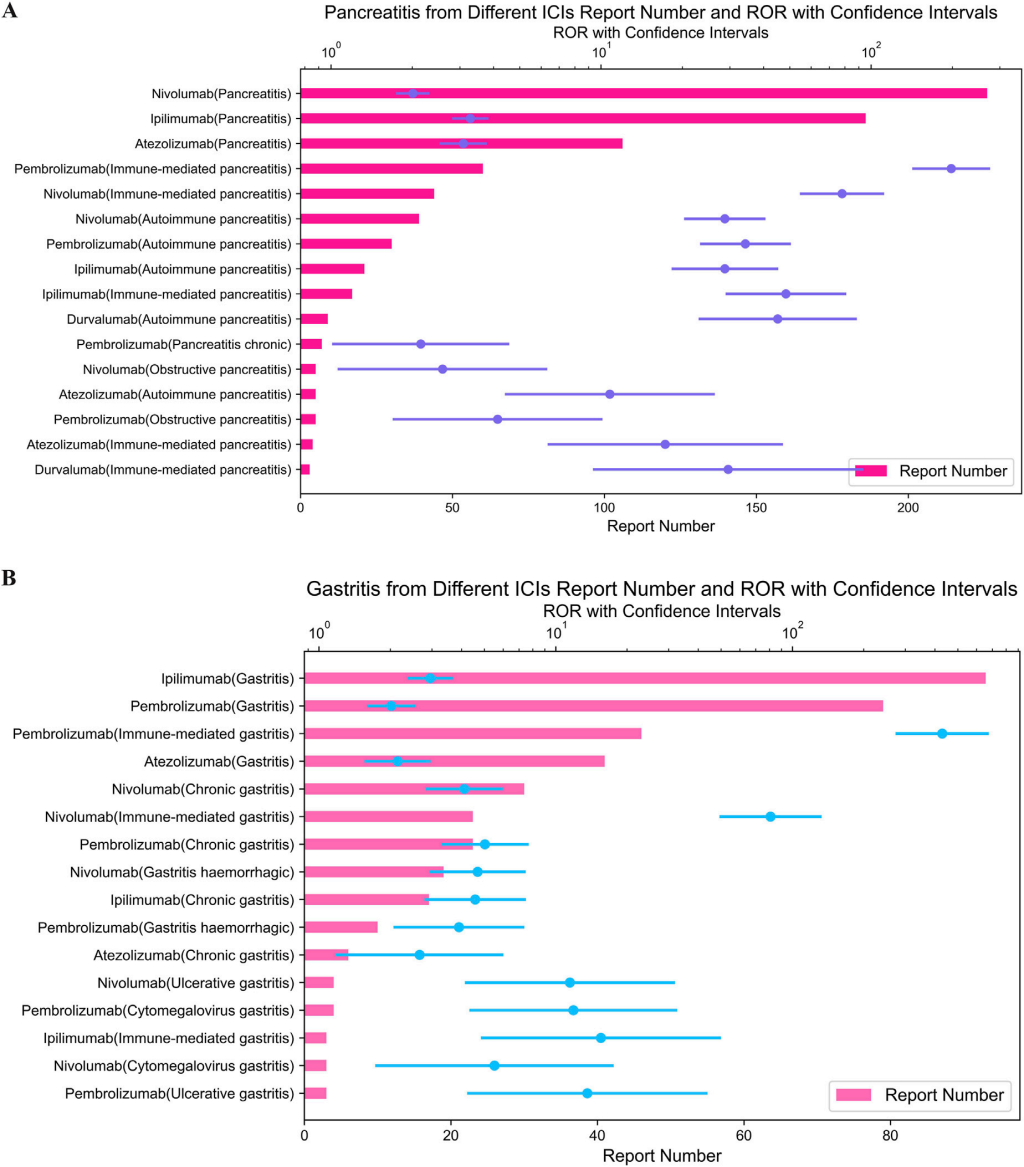
**(A)** Signals with hepatitis from different ICI. **(B)** Signals with pancreatitis from different ICI.

Pembrolizumab also showed the most highest signal for immune-mediated cholangitis (ROR = 249.18; 95% CI: 169.04–367.32) (Figure 3B), hepatitis (ROR = 85.51; 95% CI: 73.22–99.86) (Figure 4A), and pancreatitis (ROR = 198.08; 95% CI: 143.72–273.01) (Figure 4B) among the five ICIs.

**FIGURE 5:**
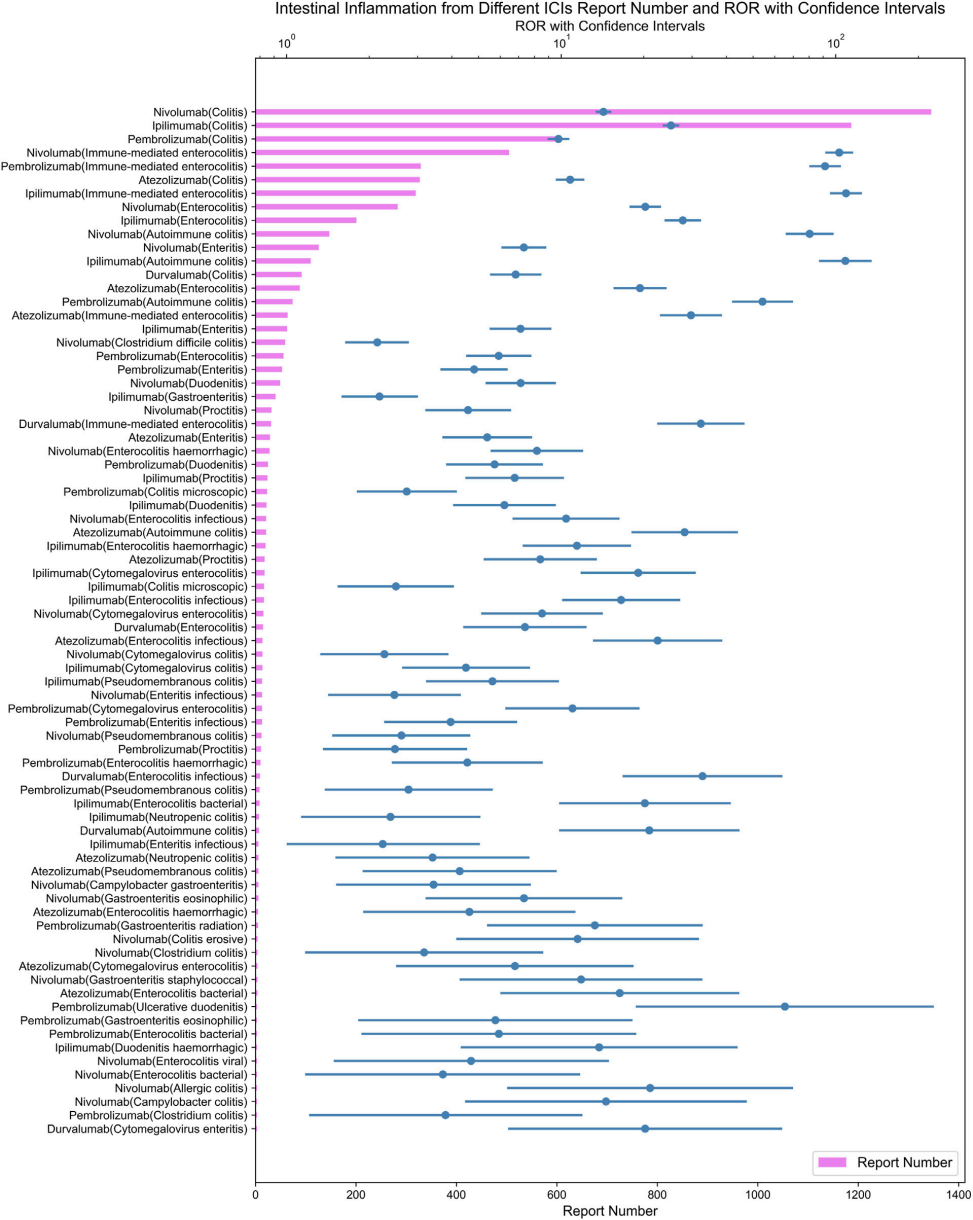
Signals with intestinal inflammation from different ICI.

As demonstrated in Figure 5, ipilimumab, an CTLA-4 inhibitor, exhibited the strongest signal for immune-mediated enterocolitis (ROR = 108.97; 95% CI: 96.26–123.37) among ICIs in our analysis.

It is worth noting that we also performed a proportional reporting rate (PRR) analysis, which confirmed all signals identified by the ROR method (Some important data are attached in Supplementary Table S2 (Supplementary Material 1)).

### 3.2 Systematic review

**FIGURE 6:**
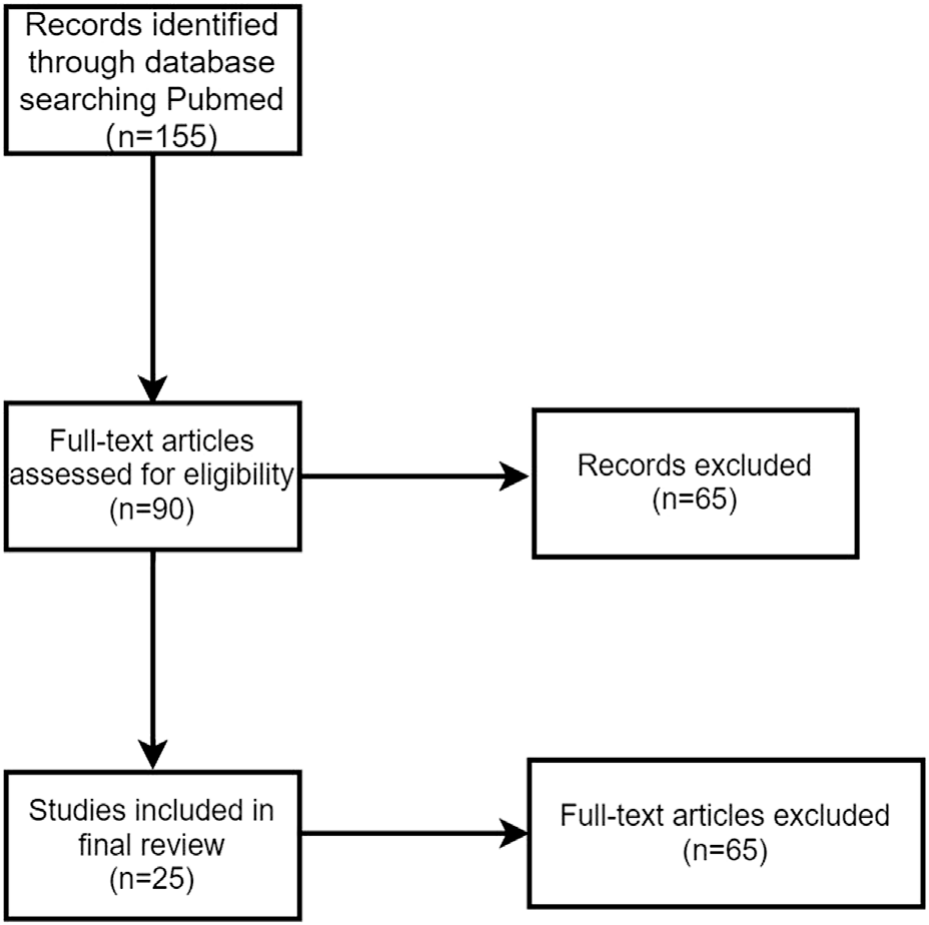
Flowchart of the systematic review process.

Our systematic literature search identified 155 relevant articles through PubMed (Figure 6). After screening, 25 case reports and case series met our inclusion criteria (Cappelli et al., 2016; Calabrese et al., 2017; Teyssonneau et al., 2017; Le Burel et al., 2017a; Ghosn et al., 2018; Takahashi et al., 2018; Javier et al., 2018; Ramos-Casals et al., 2019; Warner et al., 2019; Glick et al., 2020; Ortiz Brugués et al., 2020; Higashi et al., 2020; Pringle et al., 2020; Katsura et al., 2021; Flores et al., 2021; Njonnou et al., 2022; Ichihara et al., 2023; Wei et al., 2023; Segawa et al., 2023; Caeyman et al., 2023; Kudo et al., 2024; Kumagai et al., 2024; Pellegrino et al., 2024; Baron, 2025; Usui et al., 2025), from which we extracted data on 93 cases of PD-1/PD-L1 inhibitor-induced or PD-1/CTLA-4 combination-induced Sjögren’s syndrome/sialadenitis.

The demographic and clinical characteristics of the patients are summarized in Supplementary Table S3 (Supplementary Material 1). The study population had a median age of 62 years (range: 21–79 years). Regarding treatment regimens, PD-1 inhibitors alone were used in 49 cases (53%), while combination therapy with PD-1 and CTLA-4 inhibitors was administered in 20 cases (22%). The most common underlying malignancies were melanoma (n = 30, 32%) and lung cancer (n = 30, 32%).

For management of salivary gland involvement, 37 patients (40%) received targeted therapies including intravenous immunoglobulin (n = 3, 3%), while 31 patients (33%) were treated with symptomatic systemic therapies. One mortality was reported (1%), though the causal relationship with sialadenitis could not be definitively established.

## 4 Discussion

### 4.1 Upper gastrointestinal tract

ICIs-related upper Gastrointestinal Tract (GI) irAEs, including esophagitis and gastritis, exhibit distinct patterns across ICI classes and require targeted clinical attention.

#### 4.1.1 Oesophagitis

ICIs-related oesophagitis is relatively rare, with only sporadic case reports in existing literature (Kudo et al., 2024). Our pharmacovigilance analysis identified stronger association signals for immune-mediated esophagitis with anti-PD-1 inhibitors (nivolumab and pembrolizumab) compared to other ICI classes—to our knowledge, this is the first study to highlight this specific correlation. However, due to the small number of reported cases for these drug-event combinations, the confidence intervals of effect estimates remain wide, and these signals should be interpreted as hypothesis-generating rather than definitive conclusions.

Retrospective evidence supports this trend: in a study of 21 patients with ICI-induced oesophagitis, 15 (71%) received anti-PD-1/PD-L1 monotherapy, 1 (5%) received anti-CTLA-4 monotherapy alone, and 5 (24%) received combination therapy (Kumagai et al., 2024). Consistent with this, other upper GI irAEs are more frequently associated with anti-PD-1/PD-L1 agents than anti-CTLA-4 drugs (Pellegrino et al., 2024; Baron, 2025; Usui et al., 2025; Tong et al., 2025). Researchers have hypothesized that these class-specific toxicities may relate to tissue-specific expression patterns of ICI targets (Hu et al., 2020; Fu et al., 2024). Notably, the onset of upper GI irAEs differs chronologically from lower GI events: Panneerselvam et al. reported a median time of 4 months from ICI initiation to esophagitis onset (Kumagai et al., 2024), whereas lower GI irAEs such as colitis typically manifest within 6–8 weeks (Farha et al., 2023). This delayed presentation emphasizes the need for prolonged monitoring of upper GI symptoms beyond the initial treatment phase.

#### 4.1.2 Gastritis

Our analysis revealed a stronger association between pembrolizumab and immune-mediated gastritis compared to other ICIs. This aligns with a tertiary care center study by Farha et al., which documented 10 cases of ICI-associated gastritis caused by pembrolizumab among 25 total cases (Rabbani et al., 2024). Additionally, high-dose, short-interval administration of pembrolizumab has been linked to increased incidence of nausea and vomiting (Meunier et al., 2024), suggesting a potential dose-dependent relationship for gastritis risk. Importantly, ICI-associated gastritis may occur independently or, more commonly, coexist with enteritis/colitis—a clinical distinction that can guide diagnostic workup and management.

Across all upper GI irAEs, the broad immune-activating mechanism of ICIs explains their occurrence across multiple ICI classes (consistent with potential “category effects”), but precise risk differences between agents require validation in larger cohorts.

### 4.2 Hepatobiliary system

Hepatobiliary irAEs (hepatitis, cholangitis, and cholecystitis) represent critical safety concerns in ICI therapy, with varying incidence and ICI-class specificity.

#### 4.2.1 Hepatitis

Hepatitis is one of the most common digestive system irAEs associated with ICIs. Our analysis found that anti-PD-1 inhibitors (pembrolizumab and nivolumab) exhibited stronger signals for immune-mediated hepatitis—a result consistent with Fu et al.‘s pharmacovigilance study (Baraibar et al., 2019). This contrasts with anti-CTLA-4 agents (e.g., ipilimumab), which show weaker hepatitis signals but higher propensity for other irAEs (e.g., colitis).

The clinical significance of ICI-related hepatitis is underscored by its contribution to fatal irAEs: a meta-analysis and subsequent research revealed that among 333 anti-PD-1/PD-L1-related deaths, hepatitis accounted for 22.5% (75 cases), second only to pneumonitis (Wolchok et al., 2010; Hodi et al., 2016). Timely intervention is critical, as untreated immune-mediated hepatitis can progress to life-threatening liver failure (Jiang et al., 2019; Chang et al., 2020; Stamatouli et al., 2024; Abu-Sbeih et al., 2019).

#### 4.2.2 Cholangitis

Gender does not significantly affect the incidence of ICI-related cholangitis (Quandt et al., 2020), but ICI class specificity is pronounced. Meunier et al. reported 48 cases of ICI-related cholangitis, 41 of which were associated with anti-PD-1 inhibitors (Liu et al., 2021)——a finding supported by our real-world data, which demonstrated significantly stronger immune-mediated cholangitis signals for nivolumab and pembrolizumab compared to anti-PD-L1 agents (e.g., atezolizumab). This aligns with previous case reports linking nivolumab and pembrolizumab to cholangitis (Hellmann et al., 2018; Martins et al., 2019; Raschi et al., 2019; Oh et al., 2017; Mollica et al., 2022; Shatila et al., 2024; Panneerselvam et al., 2021), including rare but severe presentations such as secondary sclerosing cholangitis (Hellmann et al., 2018).

#### 4.2.3 Cholecystitis

The overall incidence of ICI-induced cholecystitis is approximately 0.6%, but class-specific risk remains controversial. Abu-Sbeih et al. observed higher cholecystitis risk with anti-CTLA-4 monotherapy or combination therapy (40% of 25 cases) compared to anti-PD-1/PD-L1 monotherapy (60%) (Boike and Dejulio, 2017). In contrast, our data identified stronger cholecystitis signals for the anti-PD-L1 inhibitor atezolizumab. These discrepancies may stem from differences in study populations, drug mechanisms, or methodological approaches (e.g., spontaneous reporting vs retrospective cohorts). Large-scale, prospective studies are needed to resolve this inconsistency.

### 4.3 Mechanism of PD-1 inhibitor-associated upper GI and hepatobiliary toxicity

The stronger association of PD-1 inhibitors with upper gastrointestinal and hepatobiliary inflammation primarily stems from the critical role of the PD-1/PD-L1 pathway in maintaining local immune homeostasis within these organs. Unlike CTLA-4, which primarily acts on T-cell activation in lymph nodes, PD-1/PD-L1 signaling is a core mechanism for maintaining peripheral tolerance. In the upper gastrointestinal tract, the esophageal and gastric mucosal epithelium constitutively expresses PD-L1 to suppress abnormal immune responses to dietary and microbial antigens, and PD-1 inhibitors disrupt this protective barrier. In the liver, the high expression of PD-L1 on hepatocytes and cholangiocytes is crucial for maintaining immune tolerance in this organ, which is constantly exposed to gut-derived antigens. PD-1 inhibitors release the suppression on tissue-resident memory T cells, leading to hepatitis and cholangitis. These differences in tissue-specific target expression and biological function collectively determine the unique organ toxicity profile of PD-1 inhibitors.

### 4.4 Lower gastrointestinal tract

Colitis is the most common lower GI irAE and a hallmark toxicity of anti-CTLA-4 therapy, with clear class-specific patterns and clinical implications.

#### 4.4.1 Incidence and ICI class specificity

Our analysis confirmed that colitis is a dominant digestive irAE, with ipilimumab (anti-CTLA-4) exhibiting significantly stronger signal intensities for colitis compared to anti-PD-1/PD-L1 agents—consistent with previous systematic reviews (Onuki et al., 2018). This specificity is mechanistically driven: ipilimumab blocks CTLA-4/B7 interactions, enhancing T-cell activation and proliferation (Acero Brand et al., 2018). The intestinal mucosa, rich in immune-active cells (e.g., T cells and dendritic cells), is particularly sensitive to this immunostimulation, leading to immune-mediated tissue damage (Horisberger et al., 2018).

Gender modulates colitis risk: current studies show that ICI-related colitis primarily affects male patients (Wang DY. et al., 2017), and our findings support the notion that male patients are more susceptible to ICI-related lower GI irAEs. Geographical variations also exist, with North American populations demonstrating a more prominent risk for digestive system irAEs (including colitis) compared to other continents.

#### 4.4.2 Clinical course and management

Colitis typically manifests within 6–8 weeks of ICI initiation (Farha et al., 2023), earlier than upper GI irAEs. It is also a leading cause of fatal ICI-related adverse events: among 193 anti-CTLA-4-related deaths, colitis accounted for 70.0% (135 cases) (Wolchok et al., 2010; Hodi et al., 2016). A dose-dependent relationship further characterizes this toxicity: randomized controlled trials show that ipilimumab dose correlates with colitis incidence and severity (Wang PF. et al., 2017), and higher ICI doses (especially in anti-CTLA-4 monotherapy or anti-PD-1/PD-L1 + anti-CTLA-4 combination therapy) are positively associated with lower GI irAE risk (Hong et al., 2024; Elad et al., 2022; Le Burel et al., 2017b; Fisher et al., 2017).

Given these risks, regular monitoring for colitis symptoms (e.g., diarrhea, abdominal pain) during the first 8–12 weeks of ICI therapy is recommended (Christodoulou et al., 2010), with prompt intervention to avoid treatment interruption and severe complications.

### 4.5 Salivary glands

ICIs-related salivary gland toxicities, including sialadenitis and xerostomia, are often underrecognized but substantially impact patient quality of life.

#### 4.5.1 Sialadenitis and xerostomia

Our analysis identified an association signal between ICIs (especially nivolumab) and sialadenitis—a finding relevant given the high prevalence of oral mucosal inflammation in ICI-treated patients (1.5%–6.3%, with 0.2% experiencing severe cases) (Zhang et al., 2022). Xerostomia (dry mouth) is a common manifestation, with an incidence of 0.3% in patients receiving anti-PD-1/PD-L1 monotherapy and 2.5% in those on combination therapy with anti-CTLA-4 agents (ZhangT et al., 2022). Atypical oral manifestations (e.g., taste disturbances) occur in up to 5% of patients, sometimes indicating ICI-induced Sjögren’s syndrome (Zhang et al., 2022).

Histopathologically, ICI-induced xerostomia differs from idiopathic Sjögren’s syndrome: lip gland biopsies show mild chronic sialadenitis or focal lymphocytic sialadenitis, with predominant T-cell infiltration and minimal B-cell involvement (Patnaik et al., 2015). The pathogenesis involves disruption of the PD-1/PD-L1 pathway by ICIs, which activates T lymphocytes and induces salivary gland epithelial cell infiltration (Yoshikawa et al., 2021). While not life-threatening, xerostomia impairs taste, disrupts eating habits, and increases infection risk—highlighting the need for supportive care.

#### 4.5.2 Clinical correlates

Notably, Sjögren’s syndrome induced by ICIs is closely linked to treatment efficacy (Warner et al., 2019; Higashi et al., 2020), suggesting a potential association between immune activation (therapeutic effect) and salivary gland toxicity. Sialadenitis and xerostomia also exhibit class-specific patterns: toxicities are more pronounced with anti-CTLA-4 agents and combination therapy (ZhangT et al., 2022), though our data identified nivolumab (anti-PD-1) as a key associated agent.

### 4.6 Future research directions

To address gaps in current knowledge, we recommend future studies focus on three areas:

a. Pathological Characterization: Using tissue biopsies to define the pathological features of ICI-associated esophagitis and sialadenitis, which could clarify class-specific mechanisms.
b. Target Expression Analysis: Evaluating PD-1/PD-L1 pathway expression patterns in the esophagus and salivary glands to explain tissue-specific toxicity.
c. Causality Verification: Establishing animal models to confirm the causal relationship between specific ICIs and rare digestive irAEs (e.g., atezolizumab-related cholecystitis) (Yamano et al., 2024; Gelsomino et al., 2016; Gelsomino et al., 2018; Kawakami et al., 2017).

## 5 Study limitations

While the findings of this study hold significant clinical implications and can provide valuable references for clinical decision-making regarding immune checkpoint inhibitors (ICI), the following limitations should be noted: First, as the FAERS database is a spontaneous reporting system, it is subject to several inherent limitations including under-reporting, reporting biases (such as media attention bias or notoriety bias), and incomplete clinical information. These factors may lead to overestimation or underestimation of certain associations. Second, the lack of detailed clinical patient data (e.g., drug dosage, treatment history, concomitant medications, underlying comorbidities, and disease stage) limits our ability to conduct adjusted analyses or control for potential confounders. For instance, prior chemotherapy/radiotherapy, autoimmune comorbidities, concomitant medications such as antibiotics/PPIs, cancer stage may significantly influence the occurrence and severity of irAEs but could not be accounted for in this study. Recent studies have highlighted that comedications, particularly antibiotics and proton pump inhibitors, may modulate the gut microbiota and immune microenvironment, thereby altering the risk and clinical presentation of irAEs (Lasagna et al., 2023; Okamoto et al., 2025; Nara et al., 2024). Additionally, the database does not allow for accurate determination of incidence rates or direct comparison of absolute risks between different ICIs. Finally, the study could not accurately distinguish the specific disease contexts in which irAEs occurred. Given that ICI are approved in the U.S. for multiple cancer indications, different tumor types and their associated treatments may significantly influence the incidence and clinical manifestations of irAEs.

To mitigate the inherent limitations of disproportionality analysis using FAERS data, we implemented the following methodological optimizations:a)Strict data cleaning procedures, including deduplication, standardized terminology, and outlier handling, to ensure data quality. b)A conservative signal detection threshold (ROR ≥2 with ≥3 reported cases) combined with 95% confidence intervals to improve specificity. c)Systematic comparison of detected disproportionality signals with existing clinical evidence, drug labels, and case reports to validate clinical relevance. Future research should further validate these findings through multicenter prospective studies and explore the underlying mechanisms and risk factors of irAEs in greater depth.

## 6 Conclusion

While self-reported data inherently have limitations and ICI-associated pancreatitis and colitis have been extensively documented in the FAERS database, our analysis of FAERS data has identified noteworthy new safety signals warranting further investigation. The irAEs observed in our study, such as sialadenitis and oesophagitis, compensate for the lack of attention paid to ICI-related upper GI irAEs in previous FAERS studies, while demonstrating the value of pharmacovigilance databases in hypothesis generation. These findings add fresh dimensions to the evidence-based framework for ICI safety profiles, potentially informing clinical surveillance strategies. Given the rapid advancements in related research in recent years, timely updates to current guidelines to reflect these latest findings will hold significant reference value.

## Data Availability

The original contributions presented in the study are included in the article/Supplementary Material, further inquiries can be directed to the corresponding author.
