# Supplementary material for: Association between immune check point inhibitors and digestive system inflammatory adverse reactions: evidence from pharmacovigilance analysis and systematic review
Source: Front Pharmacol. 2025 Oct 27;16:1684475. doi: 10.3389/fphar.2025.1684475 (PMC12598047; doi:10.3389/fphar.2025.1684475)
Supplement: Supplementary file 1 [file Supplementaryfile1.docx]

***Supplementary Material***

**Associations between different ICI and adverse reactions to digestive system inflammation: A pharmacovigilance study and systematic review**

Ya Zou^1†^, Qinchuan Li^1†^, Lu Zhou^1^, Yun Lu^1^, Hua Wei^1^, Yan Zhou^1^, Shibo Lin^1^, Xirui Guo^1^, Shihao Yan^1^, Hongju Wang^1^, Fangqing Xie^1^, Chun Liu^1^, Li Chen*^2^

^1^Department of Clinical Pharmacology, Chengdu Second People's Hospital, Chengdu 610021, China;

^2^Department of Pharmacy and Evidence Based Pharmacy Center, West China Second University Hospital, Sichuan University, Chengdu, China Department of Pharmacology, Faculty of Medicine, University of the Basque Country, UPV/EHU, Leioa, Spain.

*Correspondence: Li Chen. email: [chenl_hxey@scu.edu.cn](mailto:chenl_hxey@scu.edu.cn)

† These authors have contributed equally to this work.

| Event of interest | MedDRA PT |
| --- | --- |
| Sialoadenitis  Oesophagitis | “Sialoadenitis”  “Oesophagitis”,”Oesophagitis ulcerative”, “Immune-mediated oesophagitis” |
| Cholecystitis  Cholangitis | “Cholecystitis”, “Cholecystitis acute”  “Cholangitis”, “Cholangitis acute”, “Cholangitis sclerosing”, “Immune-mediated cholangitis”, “Autoimmune cholangitis” |
| Hepatitis | “Hepatitis”, “Autoimmune hepatitis”, “Hepatitis alcoholic”,  “Hepatitis cholestatic”, “Hepatitis fulminant”, “Hepatitis toxic”,  “Immune-mediated hepatitis” |
| peritonitis | “Spontaneous bacterial peritonitis” |
| pancreatitis | “Autoimmune pancreatitis”, “Immune-mediated pancreatitis”,  “Obstructive pancreatitis”, “Pancreatitis chronic” |
| Gastritis | “Gastritis”, “Chronic gastritis”, “Cytomegalovirus gastritis”,  “Gastritis haemorrhagic”, “Immune-mediated gastritis”,  “Ulcerative gastritis” |
| Enteritis | “Autoimmune colitis”, “Clostridium colitis”, “Colitis”,  “Colitis microscopic”, “Cytomegalovirus enterocolitis”,  “Duodenitis”, “Enteritis”, “Enteritis infectious”, “Enterocolitis”, “Enterocolitis bacterial”, “Enterocolitis haemorrhagic”, “Gastroenteritis eosinophilic”,  “Gastroenteritis radiation”, “Immune-mediated enterocolitis”, “Proctitis”,  “Pseudomembranous colitis”, “Ulcerative duodenitis” |

**Table S1.** Summary of major scopes used for signal detection

**Table S2.**Search strategy in PubMed

| Database | Search Details | Results |
| --- | --- | --- |
| **PubMed** | #1:(("Immune Checkpoint Inhibitors"[Mesh] OR "Immune Checkpoint Inhibitors"[Pharmacological Action] OR "Programmed Cell Death 1 Receptor/antagonists and inhibitors"[Mesh] OR "CTLA-4 Antigen/antagonists and inhibitors"[Mesh] OR "PD-1 inhibitor*"[tiab] OR "PD-L1 inhibitor*"[tiab] OR "CTLA-4 inhibitor*"[tiab] OR "immune checkpoint inhibitor*"[tiab] OR "ICI"[tiab] OR "ipilimumab"[tiab] OR "nivolumab"[tiab] OR "pembrolizumab"[tiab] OR "atezolizumab"[tiab] OR "durvalumab"[tiab] OR "avelumab"[tiab] OR "cemiplimab"[tiab])) | 76417 |
|  | #2:(("Sjogren's Syndrome"[Mesh] OR "Sialadenitis"[Mesh] OR "Xerostomia"[Mesh] OR "sicca syndrome"[tiab] OR "Sjogren*"[tiab] OR "salivary gland inflammation"[tiab] OR "sialadenitis"[tiab] OR "parotitis"[tiab] OR "dry mouth"[tiab] OR "xerostomia"[tiab] OR "salivary dysfunction"[tiab])) | 41104 |
|  | #3:#1 AND #2 | 155 |

**Table S3.** The characteristics of ICIs associated irAEs

| **Characteristics** | **All ICIs**  **N=11398** | **Pembrolizumab**  **N=2681** | **Atezdizumab**  **N=1353** | **Durvalumab**  **N=395** | **Nvolumab**  **N=5046** | **Ipilimumab**  **N=1923** |
| --- | --- | --- | --- | --- | --- | --- |
| **Demographics** |  |  |  |  |  |  |
| **Sex(%)** |  |  |  |  |  |  |
| **Female** | 3954(34.69%) | 1134(42.30%) | 419(30.97%) | 149(37.72%) | 1676(33.21%) | 576(29.95%) |
| **Male** | 6191(54.32%) | 1442(53.79%) | 636(47.01%) | 208(52.66%) | 2889(57.25%) | 1016(52.83%) |
| **Unknown** | 1253(10.99%) | 105(3.92%) | 298(22.03%) | 38(9.62%) | 481(9.53%) | 331(17.21%) |
| **Age group(%)** |  |  |  |  |  |  |
| **＜18** | 408(3.58%) | 127(4.74%) | 90(6.05%) | - | 143(2.83%) | 48(12.50%) |
| **18-44** | 674(5.91%) | 125(4.66%) | 48(3.55%) | 12(3.04%) | 343(6.80%) | 146(7.59%) |
| **45-64** | 3331(29.22%) | 718(26.78%) | 335(24.76%) | 119(30.13%) | 1627(32.24%) | 532(27.67%) |
| **65-74** | 2968(26.04%) | 751(28.01%) | 334(24.69%) | 126(31.90%) | 1352(26.79%) | 405(21.06%) |
| **≥75** | 1633(14.33%) | 456(17.01%) | 174(12.86%) | 53(13.42%) | 682(13.52%) | 268(13.94%) |
| **Unknown** | 2384(20.92%) | 504(18.80%) | 372(27.49%) | 85(21.52%) | 899(17.82%) | 524(27.25%) |
| **Reporter(%)** |  |  |  |  |  |  |
| **Physician** | 5466(47.96%) | 1270(47.37%) | 1049(77.53%) | 312(78.99%) | 2155(42.71%) | 680(35.36%) |
| **Pharmacist** | 605(5.31%) | 176(6.56%) | 52(3.84%) | 8(2.03%) | 265(5.85%) | 104(5.41%) |
| **Lawyer** | 6(0.05%) | 2(0.07%) | - | - | 4(0.08%) | - |
| **Consumer** | 1556(13.65%) | 755(28.16%) | 33(2.44%) | 11(2.78%) | 516(10.23%) | 241(12.53%) |
| **Other health-professional** | 1821(15.98%) | 168(6.27%) | 17(1.26%) | 6(1.52%) | 1012(20.06%) | 618(32.14%) |
| **health-professional** | 1886(16.55%) | 301(11.23%) | 200(14.78%) | 24(6.08%) | 1089(21.58%) | 272(14.14%) |
| **Unknown** | 58(0.51%) | 9(0.34%) | 2(0.15%) | 34(8.61%) | 5(0.10%) | 8(0.42%) |
| **Reporter year(%)** |  |  |  |  |  |  |
| **2015** | 421(3.69%) | 56(2.09%) | - | - | 141(2.79%) | 224(11.65%) |
| **2016** | 569(4.99%) | 67(2.50%) | 11(0.81%) | - | 295(5.85%) | 196(10.19%) |
| **2017** | 999(8.76%) | 158(5.81%) | 46(3.40%) | 24(6.08%) | 508(10.07%) | 263(13.68%) |
| **2018** | 1258(11.04%) | 279(10.41%) | 86(6.36%) | 69(17.47%) | 621(12.31%) | 203(10.56%) |
| **2019** | 1615(14.17%) | 344(12.83%) | 136(10.05%) | 62(15.70%) | 825(16.35%) | 248(12.90%) |
| **2020** | 1609(14.12%) | 358(13.35%) | 175(12.93%) | 60(15.19%) | 797(15.79%) | 219(11.39%) |
| **2021** | 1759(15.43%) | 427(15.93%) | 314(23.21%) | 60(15.19%) | 806(15.97%) | 152(7.90%) |
| **2022** | 1629(14.29%) | 451(16.82%) | 299(22.10%) | 51(12.91%) | 617(12.23%) | 211(10.97%) |
| **2023** | 1538(13.49%) | 541(20.18%) | 286(21.14%) | 69(17.47%) | 436(8.64%) | 206(10.71%) |
| **Unknown** | 1(0.01%) | - | - | - | - | 1(0.05%) |
| **Country(%)** |  |  |  |  |  |  |
| **Asian** | 3582(31.43%) | 974(36.33%) | 611(45.16%) | 148(37.47%) | 1412(27.98%) | 437(22.72%) |
| **European** | 3450(30.27%) | 774(28.87%) | 374(27.64%) | 97(24.56%) | 1700(33.69%) | 505(26.26%) |
| **South America** | 112(0.98%) | 27(1.01%) | 21(1.55%) | 6(1.52%) | 50(0.99%) | 8(0.42%) |
| **North America** | 3916(34.36%) | 844(31.48%) | 323(23.87%) | 132(33.42%) | 1703(33.75%) | 914(47.53%) |
| **Oceania** | 314(2.75%) | 56(2.09%) | 22(1.63%) | 11(2.78%) | 169(3.35%) | 56(2.91%) |
| **Africa** | 13(0.11%) | 4(0.15%) | - | 1(0.25%) | 6(0.12%) | 2(0.10%) |
| **Unknown** | 11(0.10%) | 2(0.07%) | 2(0.15%) | - | 6(0.12%) | 1(0.05%) |

**Table S4.**Characteristics, treatment, and outcomes of reported cases of ICI-induced Sicca syndrome/ Sialadenitis

| Author | Age | Sex | Primary Cancer | ICI used | Time-to-onset（Months） | Managemnet | Outcome |
| --- | --- | --- | --- | --- | --- | --- | --- |
| Cappelli(2017) | 61 | Male | Non-small cell lung cancer | Nivolumab | 2.8 | CS | SD |
|  | 57 | Male | Melanoma | Nivolumab plus Ipilimumab | 3.2 | CS | PD |
|  | 74 | Male | Melanoma | Ipilimumab | ~18.0* | CS | PR |
|  | 74 | Female | Melanoma | Nivolumab | 8.0 | CS | PD |
| Calabrese(2017) | 57 | Female | Melanoma | Nivolumab plus Ipilimumab | 1.5 | CS | PR |
|  | 61 | Male | Melanoma | Nivolumab plus Ipilimumab | 1.2 | CS | PR |
|  | 63 | Male | RCC | Atezolizumab | 5.0 | CS | PR |
|  | 68 | Male | Melanoma | Nivolumab plus Ipilimumab | 1.9 | CS | PR |
| Teyssonneau(2017) | 36 | Female | left parotid acinic cell  carcinoma | Pembrolizumab | 9.8 | CS | SD |
| Burel(2017) | 56 | Female | RCC | PD-1 plus CTLA-4 | 2.4 | No | PR |
|  | 76 | Female | Urothelial bladder  cancer | PD-L1 | 0.5 | No | PR |
|  | 58 | Female | Cervical  squamous cell  carcinoma | PD-1 | 1.4 | No | SD |
|  | 60 | Male | Melanoma | PD-L1 | 1.7 | CS | PR |
| Ghosn(2018) | 69 | Female | acral lentiginous melanoma | Pembrolizumab | 8 | CS,IVIG | NR |
| Takahashi（2018） | 70 | Male | pulmonary adenocarcinoma | Pembrolizumab | 4.0 | CS | SD |
| Narváez（2018） | 66 | Female | Pancreatic  neuroendocrine  cancer | Nivolumab plus Ipilimumab | 3.5 | CS | SD |
|  | 63 | Male | Adeno carcinoma of the lung | Nivolumab | 6.0 | No | SD |
| Ramos-Casals（2019） | 61 | Female | Lung cancer | Nivolumab | 10 | No | SD |
|  | 72 | Female | Lung cancer | Nivolumab plus Ipilimumab | 7 | CS | N/A |

**Table S4.**(continued)

| Author | Age | Sex | Primary Cancer | ICI used | Time-to-onset（Months） | Managemnet | Outcome |
| --- | --- | --- | --- | --- | --- | --- | --- |
|  | 39 | Male | Melanoma | Nivolumab plus Ipilimumab | 1 | CS，MMF | N/A |
|  | 52 | Female | Colon cancer | Nivolumab plus Ipilimumab | 1 | CS | N/A |
|  | 79 | Female | Lung cancer | Pembrolizumab | 1 | CS,IVIG | N/A |
|  | 71 | Female | Melanoma | Nivolumab | 6 | CS,TAC | N/A |
|  | 64 | Male | Lung cancer | Durvalumab | 1 | CS,MMF,IVIG | N/A |
|  | 71 | Male | Lung cancer | Pembrolizumab | 7 | CS | N/A |
|  | 79 | Male | Melanoma | Pembrolizumab | 28 | CS | N/A |
|  | 50 | Female | Lung cancer | Pembrolizumab | 5 | CS，HCQ | N/A |
|  | 74 | Male | Renal cancer | Nivolumab | 11.2 | CS | N/A |
|  | 77 | Male | Renal cancer | Nivolumab | 5.7 | No | N/A |
|  | 72 | Male | Lung cancer | Pembrolizumab | 16 | No | N/A |
|  | 70 | Male | Lung cancer | Nivolumab | 18 | No | N/A |
|  | 51 | Female | Chordoma | Durvalumab | 3.7 | No | N/A |
|  | 71 | Male | Lung cancer | Nivolumab | 2 | No | N/A |
|  | 58 | Male | Lung cancer | Pembrolizumab | 6 | No | N/A |
|  | 60 | Male | Melanoma | Durvalumab | 2 | CS | N/A |
|  | 71 | Male | Renal cancer | Nivolumab | 8.3 | CS | N/A |
|  | 68 | Female | Lung cancer | Nivolumab | 6 | No | N/A |
|  | 51 | Male | Lung cancer | Durvalumab | 3 | No | N/A |
|  | 49 | Male | Renal cancer | Nivolumab plus Ipilimumab | 4.6 | No | N/A |
|  | 56 | Female | Renal cancer | Nivolumab plus Ipilimumab | 2.4 | No | N/A |
|  | 58 | Female | Cervix cancer | Nivolumab | 2.6 | No | N/A |
|  | 67 | Male | Renal cancer | Pembrolizumab | 14 | No | N/A |
|  | 62 | Female | Renal cancer | Nivolumab | 10.5 | CS,HCQ | N/A |
| WARNER（2019） | 56 | Male | Metastatic melanoma | Nivolumab plus Ipilimumab | 1.3 | N/A | PD |
|  | 78 | Male | Metastatic melanoma | Pembrolizumab | 4.0 | N/A | Non-CR/Non-PD |
|  | 40 | Female | Metastatic melanoma | Nivolumab plus Ipilimumab | 1.5 | N/A | PD |
|  | 65 | Male | Metastatic melanoma | Pembrolizumab plus ipilimumab;then Pembrolizumab alone | 1.8 | N/A | CR |
|  | 70 | Male | Metastatic melanoma | Pembrolizumab | 4.1 | N/A | CR |
|  | 40 | Female | Metastatic melanoma | Nivolumab | 6.4 | N/A | SD |

**Table S4.**(continued)

| Author | Age | Sex | Primary Cancer | ICI used | Time-to-onset（Months） | Managemnet | Outcome |
| --- | --- | --- | --- | --- | --- | --- | --- |
|  | 40 | Female | Metastatic melanoma | Nivolumab plus Ipilimumab | 1.5 | N/A | PD |
|  | 65 | Male | Metastatic melanoma | Pembrolizumab plus ipilimumab;then Pembrolizumab alone | 1.8 | N/A | CR |
|  | 70 | Male | Metastatic melanoma | Pembrolizumab | 4.1 | N/A | CR |
|  | 40 | Female | Metastatic melanoma | Nivolumab | 6.4 | N/A | SD |
|  | 71 | Male | Metastatic melanoma | Nivolumab | 2.5 | N/A | Non-CR/Non-PD |
|  | 59 | Male | Metastatic melanoma | Pembrolizumab | 5.1 | N/A | CR |
|  | 76 | Female | Metastatic melanoma | Nivolumab | 6.9 | N/A | PR |
|  | 52 | Male | Metastatic melanoma | Nivolumab | 2.1 | N/A | SD |
|  | 57 | Female | Metastatic non-small cell lung carcinoma | PD-L1 | 3.4 | N/A | PR |
|  | 57 | Male | Metastatic prostate cancer | Avelumab | 1.0 | N/A | SD |
|  | 74 | Male | adenocarcinoma | Nivolumab | 2.0 | N/A | PD |
|  | 44 | Male | Metastatic thymic carcinoma | Avelumab | 3.2 | N/A | SD |
|  | 66 | Female | Metastatic thymic carcinoma | Avelumab | 3.2 | N/A | SD |
|  | 55 | Male | Metastatic thymic carcinoma | Avelumab | 1.8 | N/A | SD |
|  | 55 | Female | Recurrent respiratory papillomatosis | Avelumab | 1.9 | N/A | N/A |
|  | 55 | Male | Recurrent respiratory papillomatosis | Avelumab | 3.3 | N/A | N/A |

**Table S4.**(continued)

| Author | Age | Sex | Primary Cancer | ICI used | Time-to-onset（Months） | Managemnet | Outcome |
| --- | --- | --- | --- | --- | --- | --- | --- |
|  | 56 | Male | Recurrent respiratory papillomatosis | Avelumab | 2.1 | N/A | N/A |
|  | 26 | Male | Recurrent respiratory papillomatosis | Avelumab | 1.0 | N/A | N/A |
| Glick  (2020) | 44 | Female | squamous cell lung cancer | Durvalumab | 10.0 | CS | SD |
| Brugués (2020) | 42 | Male | Renal adenocarcinoma | PD-1 | 8.3 | No | PR |
|  | 60 | Male | Melanoma | PD-1 | 0.9 | Symptomatic measures | PD |
|  | 61 | Female | Oral squamous cell carcinoma | PD-1 | 0.9 | Symptomatic measures | PD |
|  | 78 | Female | Melanoma | PD-1 plus CTLA-4 | 1.8 | CS，Symptomatic measures | SD |
|  | 63 | Male | Oral squamous cell carcinoma | PD-1 | 1.8 | Symptomatic measures | N/A |
|  | 65 | Female | Endometrial adenocarcinoma | PD-1 | 6.4 | Symptomatic measures | PD |
|  | 71 | Female | Endometrial adenocarcinoma | PD-1 | 1.8 | Symptomatic measures | PD |
|  | 68 | Male | Renal adenocarcinoma | PD-1 | 2.8 | Symptomatic measures | PD |
|  | 76 | Female | Melanoma | PD-1 | 15.6 | Symptomatic measures | SD |
|  | 47 | Female | Melanoma | PD-1 plus CTLA-4 | 4.6 | No | PD |
|  | 39 | Female | Melanoma | PD-1 plus CTLA-4 | 0.5 | CS，Symptomatic measures | SD |
|  | 74 | Male | Non-small cell lung carcinoma | PD-1 | 3.7 | Symptomatic measures | N/A |
|  | 62 | Male | Pancreatic adenocarcinoma | PD-1 | 6.4 | Symptomatic measures | PR |
|  | 74 | Male | Melanoma | PD-1 | 2.8 | Symptomatic measures | PD |
|  | 69 | Male | Oral squamous cell carcinoma | PD-L1 | 0.9 | Symptomatic measures | SD |
| Higashi(2020) | 60 | Male | Gastric adenocarcinoma | Nivolumab | 1.0 | CS | PR |
| Pringle(2020) | 52 | Male | Non-small cell lung carcinoma | durvalumab | ~5.0 | NR | NR |
| Katsura(2021) | 56 | Male | Clear cell renal carcinoma | Nivolumab | ~2.5 | CS | NR |

**Table S4.**(continued)

| Author | Age | Sex | Primary Cancer | ICI used | Time-to-onset（Months） | Managemnet | Outcome |
| --- | --- | --- | --- | --- | --- | --- | --- |
| Conde-Flores(2021) | 77 | Male | Clear cell renal carcinoma | Nivolumab plus Ipilimumab | 3.0 | Symptomatic measures | SD |
| Njonnou(2022) | 70 | Male | Melanoma | Nivolumab | 6.0 | CS | SD |
| Ichihara(2023) | 68 | Female | lung adenocarcinoma | Pembrolizumab | 5.0 | NR | PR |
| Wei(2023) | 62 | Male | lung adenocarcinoma | camrelizumab | 0.56 | CS, MMF | PR |
| Segawa(2023) | 70 | Male | RCC | Nivolumab plus Ipilimumab | 3.0 | Symptomatic measures | PR |
| Caeyman(2023) | 71 | Female | Non-small cell lung carcinoma | Pembrolizumab | 18 | CS | PR |
| [Kudo](https://pubmed.ncbi.nlm.nih.gov/?term=Kudo+S&cauthor_id=38495967)(2024) | 75 | Male | small-cell lung cancer | atezolizumab | 6.0 | NR | CR |
| [Kumagai](https://pubmed.ncbi.nlm.nih.gov/?term=Kumagai+K&cauthor_id=38962488)(2024) | 73 | Female | lung adenocarcinoma | Atezolizumab | 2.5 | CS | SD |
| Pellegrino(2024) | 40 | Female | Triple-negative  ductal carcinoma | Atezolizumab | N/A | Cs | SD |
| Baron(2025) | 21 | Female | Metastatic melanoma | Nivolumab plus Ipilimumab | 0.13 | CS | SD |
| Usui(2025) | 64 | Male | hepatocellular carcinoma | durvalumab plus tremelimumab | 4.1 | NR | PD |

CS = corticosteroid; SD=Stable disease; PD=Progressive disease; PR=Partial response; RCC=Renal cell carcinoma; IVIG = intravenous immune globulin; MMF=Mycophenolate mofetil;

TAC= Tacrolimus; HCQ=hydroxychloroquine; CR=complete remission; Symptomatic measures=hydration, gum, oral hygiene, anetholtrithion/pilocarpine, salivary substitute; N/A=not applicable;NR=not reported

**Table S5.** Signals with esophagitis from different ICIs

| **drug** | **PT** | **a** | **b** | **c** | **d** | **ROR（95CI%）** | **PRR（χ2）** |
| --- | --- | --- | --- | --- | --- | --- | --- |
| Pembrolizumab | Immune-mediated oesophagitis | 5 | 37126 | 8 | 12116068 | 203.97（66.72-623.52） | 203.94(621.37) |
|  | Oesophagitis | 38 | 37093 | 4834 | 12111242 | 2.57（1.86-3.53） | 2.57(36.02) |
|  | Oesophagitis ulcerative | 7 | 37124 | 252 | 12115824 | 9.07（4.28-19.21) | 9.06(48.86) |
| Atezdizumab | Oesophagitis | 36 | 18077 | 4836 | 12130258 | 5.00(3.60-6.94) | 4.99(113.96) |
| Nivolumab | Eosinophilic oesophagitis | 8 | 59325 | 476 | 12093398 | 3.43(1.70-6.89) | 3.43(13.51) |
|  | Immune-mediated oesophagitis | 7 | 59326 | 6 | 12093868 | 237.83(79.92-707.71) | 237.80(761.84) |
|  | Necrotising oesophagitis | 4 | 59329 | 275 | 12093599 | 2.96(1.10-7.96) | 2.96(5.13) |
|  | Oesophagitis | 111 | 59222 | 4761 | 12089113 | 4.76(3.94-5.75) | 4.75(321.49) |
| Durvalumab | Radiation oesophagitis | 17 | 7887 | 158 | 12145145 | 165.68(100.41-273.38) | 165.33(2507.02) |
| Ipilimumab | Oesophagitis | 51 | 29987 | 4821 | 12118348 | 4.28(3.24-5.63) | 4.27(126.40) |
|  | Radiation oesophagitis | 4 | 30034 | 171 | 12122998 | 9.44(3.50-25.45) | 9.44(29.50) |

**Table S6.** Signals with gastritis from different ICIs

| **drug** | **PT** | **a** | **b** | **c** | **d** | **ROR（95CI%）** | **PRR（χ2）** |
| --- | --- | --- | --- | --- | --- | --- | --- |
| Pembrolizumab | Chronic gastritis | 23 | 37108 | 1495 | 12114581 | 5.02（3.33-7.58） | 5.02（72.93） |
|  | Cytomegalovirus gastritis | 4 | 37127 | 110 | 12115966 | 11.87（4.38-32.18） | 11.87（38.40） |
|  | Gastritis | 79 | 37052 | 12774 | 12103302 | 2.02（1.62-2.52） | 2.02（40.36） |
|  | Gastritis haemorrhagic | 10 | 37121 | 837 | 12115239 | 3.90（2.09-7.27） | 3.90（21.30） |
|  | Immune-mediated gastritis | 46 | 37085 | 35 | 12116041 | 429.39（276.61-666.55） | 428.86（8684.60） |
|  | Ulcerative gastritis | 3 | 37128 | 72 | 12116004 | 13.60（4.28-43.16） | 13.60（33.61） |
| Atezdizumab | Chronic gastritis | 6 | 18107 | 1512 | 12133582 | 2.66（1.19-5.93） | 2.66（6.18） |
|  | Gastritis | 41 | 18072 | 12812 | 12122282 | 2.15（1.58-2.92） | 2.14（24.97） |
| Nivolumab | Chronic gastritis | 30 | 59303 | 1488 | 12092386 | 4.11（2.86-5.90） | 4.11（69.20） |
|  | Cytomegalovirus gastritis | 3 | 59330 | 111 | 12093763 | 5.51（1.75-17.34） | 5.51（10.78） |
|  | Gastritis haemorrhagic | 19 | 59314 | 828 | 12093046 | 4.68（2.97-7.37） | 4.68（53.70 |
|  | Immune-mediated gastritis | 23 | 59310 | 58 | 12093816 | 80.86（49.88-131.07） | 80.83（1298.46） |
|  | Ulcerative gastritis | 4 | 59329 | 71 | 12093803 | 11.48（4.19-31.44） | 11.48（36.24） |
| Ipilimumab | Chronic gastritis | 17 | 30021 | 1501 | 12121668 | 4.57（2.83-7.38） | 4.57（46.90） |
|  | Gastritis | 93 | 29945 | 12760 | 12110409 | 2.95（2.40-3.62） | 2.94（118.44） |
|  | Immune-mediated gastritis | 3 | 30035 | 78 | 12123091 | 15.52（4.90-49.19） | 15.52（39.25） |
